# Differential Gene Expression of Mesenchymal Stromal Cell and Granulosa Cell Markers in Equine Follicular Aspirates

**DOI:** 10.1111/rda.70288

**Published:** 2026-09-30

**Authors:** María del Prado Soriano‐Campos, Carmen Cristina Muñoz‐García, Marcos Luis‐Calero, Marian del Olmo‐Ortiz, Alejandro Gallardo‐Soler, Beatriz Macías‐García, Lauro González‐Fernández

**Affiliations:** ^1^ MINVET Group, Departamento de Medicina Animal, Facultad de Veterinaria Universidad de Extremadura Cáceres Spain; ^2^ SINTREP Group, Departamento de Bioquímica y Biología Molecular y Genética, Facultad de Veterinaria Universidad de Extremadura Cáceres Spain

**Keywords:** cell passages, equine, gene expression, granulosa cells, MSC

## Abstract

Equine follicular aspirates obtained during ovum pick‐up contain a heterogeneous mixture of mesenchymal stromal cells (MSC) and granulosa cells (GC). This study evaluates how selective isolation and expansion of MSC decrease GC populations. Follicular aspirates‐derived cells were cultured up to passage four (P4) following a previously validated protocol. While freshly isolated follicular aspirates cells (FAC) displayed heterogeneous morphologies, early passages (P3‐P4) showed a uniform, spindle‐shaped phenotype compatible with MSC. Gene expression analysis by RT‐qPCR revealed a marked upregulation of mesenchymal and stemness‐associated genes (*FN1, THY1, FGF2* and *CD105*) in P4‐MSC compared with FAC, confirming enrichment of the MSC population. Conversely, GC‐specific genes (*FSHR, FOXL2* and *CYP11A1*) were significantly upregulated in FAC. These findings suggest that early passages efficiently promote selective MSC expansion while progressively eliminating GC.

## 1 Introduction

New cell‐based therapies promote the use of mesenchymal stromal cells (MSC) due to their immunomodulatory and self‐renewal properties, and convenient availability from multiple tissue sources (Yang et al. 2018). Following ovum pick‐up (OPU), oocytes are harvested and the remaining follicular aspirate contains a mixed cell population composed of MSC, granulosa cells (GC), epithelial and thecal cells, or erythrocytes, among others. Previous studies have identified follicular aspirates as a suitable source of MSC (Dzafic et al. 2014; Muñoz‐García et al. 2026). Among these initial cell populations, GC are the predominant follicular cell type and play essential roles in follicular fluid production, oocyte development, angiogenesis and steroidogenesis (Ai et al. 2019; Kostadinova et al. 2025).

Given the intrinsic cellular heterogeneity of follicular aspirates, early passages during in vitro culture are critical to selectively expand adherent MSC while excluding other cell populations such as GC. Furthermore, cell passaging has been shown to facilitate the progressive enrichment of MSC cultures from primary sources (Chen et al. 2024; Tian et al. 2024).

Therefore, in our study, reverse transcription‐quantitative PCR (RT‐qPCR) was used to analyse the expression of stemness and MSC‐specific as well as granulosa cell‐specific genes in equine freshly isolated follicular aspirates cells (FAC) compared with homogeneous follicular aspirates MSC at passage four (P4), using our previously validated isolation protocol.

## 2 Materials and Methods

### 2.1 Isolation and Culture of Follicular Aspirates‐Derived MSC

Ovarian follicular aspirates were obtained from eight different mares during OPU sessions as previously described (Luis‐Calero et al. 2024). After oocyte collection, filtered follicular aspirates were processed by centrifugation and osmotic shock, and the resulting cells were seeded as passage zero (P0) in DMEM supplemented with foetal bovine serum (FBS), antibiotics and amphotericin B (Muñoz‐García et al. 2026).

Cultures were maintained at 37°C and 5% CO_2_, with medium renewal after 24 h and every 3–4 days. Cells were cultured until approximately 80% confluence was reached, when cells were detached using 0.05% Trypsin‐EDTA, counted and re‐seeded for new expansion (first passage, P1). This procedure was repeated until 80% confluence was reached at the third passage (P3), requiring an average of 39 days (range: 33–50 days). Following this, P3 cells were cryopreserved, later thawed and expanded to passage four (P4) for subsequent analyses, such as flow cytometry characterization and trilineage differentiation assays as previously reported (data not shown) (Muñoz‐García et al. 2026).

### 2.2 RT‐qPCR Assays

For gene expression analysis, thawed P4‐MSC lines (*n* = 8) were cultured until reaching 80% confluence, detached and lysed by adding Buffer RLT according to the manufacturer's recommendations (RNeasy Mini Kit) (1.5 × 10^6^ cells per sample). FAC samples (*n* = 4) were obtained from four additional OPU sessions, including three animals not previously used for MSC isolation and one mare from which a P4‐MSC line was isolated. After harvesting, FAC samples were immediately lysed in Buffer RLT. Total RNA was extracted (RNeasy Mini Kit), treated with DNase and cDNA was synthesized (PrimeScript RT reagent kit), according to the manufacturer's instructions. For each experimental group, equal amounts (1 μg) of total RNA from each individual sample were pooled. Subsequently, 1 μg of the pooled RNA was used for reverse transcription. qPCR was performed using RealQ Plus 2× Master Mix Green low ROX (AmpliQon, Denmark) by a Thermal Cycler QuantStudio 5 Real‐Time PCR System (Thermo Fisher Scientific, MA, USA). Each sample was analysed in triplicate, and the experiment was carried out three independent times (technical replicates; *n* = 3). Relative gene expression was calculated using the *ΔΔCt* comparative method (Livak and Schmittgen 2001). Primer design was performed as previously described (Muñoz‐García et al. 2026). Gene and primer information is shown in Tables 1 and 2.

**TABLE 1:** Mesenchymal and stemness‐associated genes analysed by RT‐qPCR.

Gene	Primer sequence (5′‐3′)	Ta; bp	GenBank	References
*THY1*	F: TGCGAACTCCGCCTCTCT	60; 93	XM_001503225.5	(Rink et al. 2017)
	R: GCTTATGCCCTCGCACTTG			
*FGF2*	F: CGGCTCTACTGCAAAAACGG	60; 91	NM_001195221.1	(Muñoz‐García et al. 2026)
	R: TGATGTGAGGGTCGCTCTTC			
*CD105*	F: GACGGAAAATGTGGTCAGTAATGA	60; 101	XM_003364144.5	(Rink et al. 2017)
	R: GCGAGAGGCTCTCCGTGTT			
*FN1*	F: GGTTTTAAGCTGGGTGTGCG	60; 152	XM_001489104	(Szóstek‐Mioduchowska et al. 2019)
	R: GCATCTCTCTCCTGCCCATC			
GAPDH*	F: GTTTGTGATGGGCGTGAACC	60; 147	NM_001163856.1	(Muñoz‐García et al. 2026)
	R: TGCACTGTGGTCATGAGTCC			

*Note:* Primers used for RT‐qPCR, including GenBank accession numbers.

Abbreviations: bp, amplicon length; *CD105*, endoglin; F, forward; *FGF2*, fibroblast growth factor 2; *FN1*, fibronectin 1; GAPDH*, glyceraldehyde‐3‐phosphate dehydrogenase (reference gene); R, reverse; Ta, annealing temperature (°C); *THY1*, Thy‐1 cell surface antigen.

**TABLE 2:** Granulosa cell‐associated genes analysed by RT‐qPCR.

Gene	Primer sequence (5′‐3′)	Ta; bp	GenBank	Reference
*FSHR*	F: CTGCCAAGAGAGCAAGGTGA	60; 128	NM_001164013.2	This work
	R: TCTCCAGGTCCCCAAATCCT			
*FOXL2*	F: ACATTATCGCCAAGTTCCCGT	60; 71	XM_023621016.2	This work
	R: GAGGTTGTGGCGGATGCTAT			
*CYP11A1*	F: CCTGGTGACAATGGCTGGAT	60; 139	XM_070231900.1	This work
	R: AAACTGACTCCACGTTGCCA			
GAPDH*	F: GTTTGTGATGGGCGTGAACC	60; 147	NM_001163856.1	(Muñoz‐García et al. 2026)
	R: TGCACTGTGGTCATGAGTCC			

*Note:* Primers used for RT‐qPCR, including GenBank accession numbers.

Abbreviations: bp, amplicon length; *CYP11A1*, cytochrome P450 family 11 subfamily A member 1; F, forward; *FOXL2*, forkhead box protein L2; *FSHR*, follicle‐stimulating hormone receptor; GAPDH*, glyceraldehyde‐3‐phosphate dehydrogenase (reference gene); R, reverse; Ta, annealing temperature (°C).

### 2.3 Statistical Analysis

Statistical analyses were performed using SPSS Statistics v31.0 software. Statistical comparisons were performed using RT‐qPCR fold‐change (FC) values. Data are presented as the mean log_2_ FC ± standard deviation (SD). qPCR reactions were performed in triplicate (technical replicates), and the complete assay was independently repeated three times. Data distribution and variance were assessed using Shapiro–Wilk and Levene's tests, respectively, and group comparisons were conducted using the Student's *t*‐test or Mann–Whitney *U* test as appropriate. Significance was set at *p* < 0.05.

## 3 Results

At passage zero, cultures derived from follicular aspirates displayed a heterogeneous mixture of non‐adherent cells, large polygonal cells, rounded cells and small fibroblast‐like cells. Following medium refresh, non‐adherent cells were removed. P3‐P4 cultures predominantly exhibited a uniform elongated morphology, compatible with homogeneous MSC populations, according to previous equine reports (Rink et al. 2017) (Figure S1). Flow cytometry confirmed that P4‐MSC expressed mesenchymal markers (CD90, CD29) and lacked haematopoietic markers (CD45, CD19, MHC‐II). P4‐MSC also exhibited trilineage differentiation into adipogenic, osteogenic and chondrogenic lineages, fulfilling the minimal criteria for MSC characterization required by the International Society for Cellular Therapy (ISCT) (Dominici et al. 2006). Further details are provided in our previous study (Muñoz‐García et al. 2026).

Gene expression analysis by RT‐qPCR revealed significant upregulation of mesenchymal and stemness genes (*FN1, THY1, FGF2* and *CD105*) in P4‐MSC compared with FAC (Figure 1). Conversely, granulosa cell‐associated genes (*FSHR, FOXL2* and *CYP11A1*) were significantly upregulated in FAC relative to P4‐MSC (Figure 2), indicating the presence of GC at baseline and their progressive loss through subsequent passages.

**FIGURE 1:**
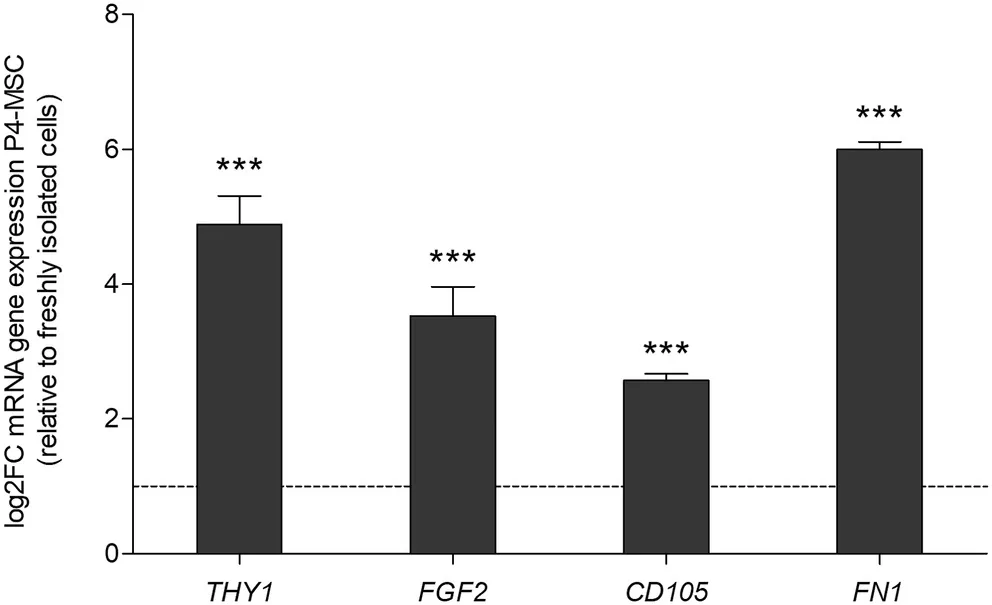
Gene expression in P4‐MSC relative to freshly isolated follicular aspirates cells. Data are presented as mean log_2_ fold change ± SD (***p < 0.001). The horizontal line represents the fold‐change threshold (log_2_ FC = 1). (*n* = 3).

**FIGURE 2:**
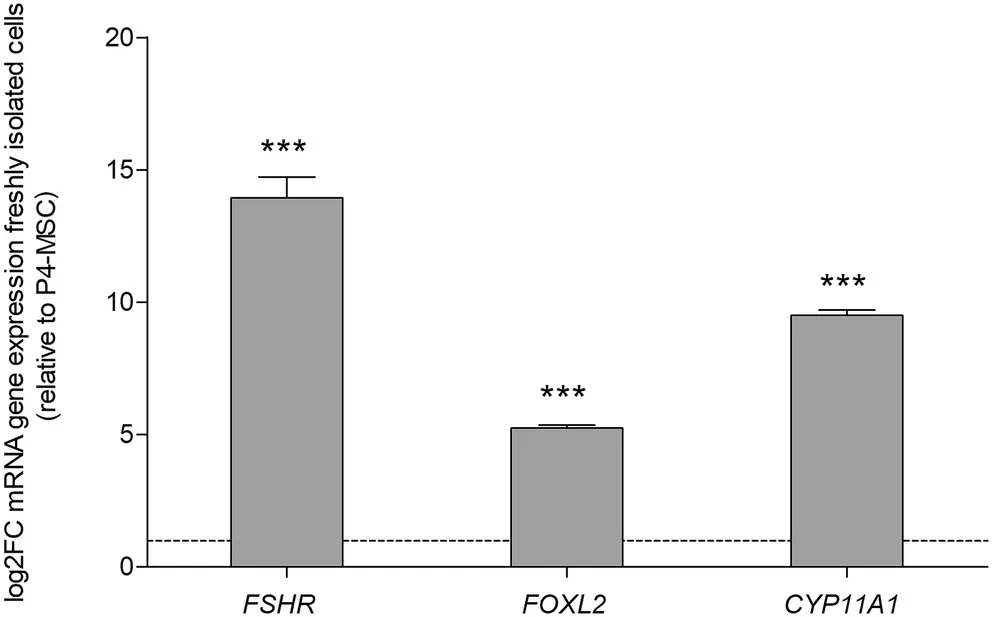
Gene expression in freshly isolated follicular aspirates cells relative to P4‐MSC. Data are presented as mean log_2_ fold change ± SD (***p < 0.001). The horizontal line represents the fold‐change threshold (log_2_ FC = 1). (*n* = 3).

## 4 Discussion

This study evaluates, by gene expression profiling, the transition from heterogeneous freshly isolated follicular aspirates cells (FAC) to homogeneous mesenchymal stromal cells (MSC) cultures at early passages (P4) in mares.

It is well described that serial passages under adherent conditions progressively enrich MSC cultures from primary cell sources, and remove non‐adherent or transient adherent cells, such as granulosa cells (GC) (Rink et al. 2017; Yang et al. 2018). In contrast to MSC, GC show limited long‐term viability when cultured in vitro under standard conditions and often require additional supplementation with specific additives such as leukaemia inhibitory factor (LIF) or follicular fluid (Ai et al. 2019; Kostadinova et al. 2025; Marin et al. 2025).

Although the proportion of GC in the initial FAC population was not directly quantified, their presence was confirmed by the upregulation of granulosa cell markers (*FSHR, FOXL2* and *CYP11A1*) relative to P4‐MSC (Ai et al. 2019; Marin et al. 2025). Meanwhile, P4‐MSC lines exhibited pronounced upregulation of *FN1, THY1, FGF2* and *CD105*, genes involved in multipotency, stemness, cell adhesion and migration, and growth factor signalling, according to previous reports (Chen et al. 2024; Yang et al. 2018). However, because the initial proportion of GC was unknown, we cannot exclude the possibility that a small proportion of non‐MSC remained in the P4 population, representing a limitation of this study. Nevertheless, our results align with previous reports, showing lower MSC‐related gene expression in freshly collected human follicular cells than in established MSC lines (Dzafic et al. 2014). Similarly, Tian et al. (2024) showed that early passages (P1–P5) are sufficient to generate stable MSC populations with enhanced stemness signatures. It should be noted that FAC and P4‐MSC samples were mostly derived from different mares, which may introduce interindividual variability in gene expression. Such variability is an inherent feature of primary cell populations and has been widely described in equine MSC studies (Harman et al. 2020). Nevertheless, the consistent gene expression trends observed between groups support the robustness of the findings.

In conclusion, early passaging (P4) effectively promotes MSC enrichment from equine follicular aspirates by removing granulosa cells, yielding homogeneous MSC cultures. These findings reinforce the suitability of follicular aspirates as a practical and accessible MSC source in horses. Our protocol allows for the obtention of homogeneous MSC populations within a relatively short culture period and could be valuable for future autologous and/or potentially allogeneic applications. However, additional in vivo studies are needed to address the functional relevance, safety and therapeutic potential of these cells.

## Author Contributions

M. P. S. ‐C., C. C. M. ‐G., M. L. ‐C. and M. O. ‐O. collected and processed the samples and contributed to the development of the experiments. M. P. S. ‐C. also analysed and interpreted the data, performed the statistical analysis and drafted the manuscript. A. G. ‐S. performed the experiments and analysed the data. B. M. ‐G. and L. G. ‐F. contributed to the study design, data analysis and interpretation, funding acquisition and the preparation and revision of the manuscript. All authors read and approved the final manuscript.

## Funding

This research was supported by PID2023‐1464930B‐I00 funded by MICIU/AEI/10.13039/501100011033. María del Prado Soriano‐Campos was funded by grant PREP2023‐001164 funded by MICIU/AEI/10.13039/501100011033 and by “ESF+”. Carmen Cristina Muñoz‐García was supported by grant CNS2023‐144173. Marcos Luis‐Calero was supported by an ‘Acción II’ grant from the ‘Plan Propio de Investigación y Transferencia de la Universidad de Extremadura’ and ‘Banco Santander.’ Marian del Olmo‐Ortiz was funded by the Extremadura Public Employment Service (SEXPE) under the “Atraigo Talento” program and co‐funded by the “ESF+”. Alejandro Gallardo‐Soler was funded by grant PID2023‐146493OB‐I00. This work has been co‐funded by the European Union, European Regional Development Fund (85%), and the Junta de Extremadura. Managing authority: Ministerio de Hacienda (Spain). Grant GR24094.

## Conflicts of Interest

The authors declare no conflicts of interest.

## Supporting information


**Figure S1:** Representative micrographs showing the morphological evolution of MSC along serial passages. (a) heterogeneous P0 culture; (b) heterogenous P1 culture; (c) spindle‐shaped P4 culture.

## Data Availability

The data that support the findings of this study are available from the corresponding author upon reasonable request.
